# The Effect of Online Education on Healthy Eyes of Saudi Teachers in the COVID-19 Pandemic: A Local Study

**DOI:** 10.7759/cureus.24721

**Published:** 2022-05-04

**Authors:** Saif K Dossari, Rahaf AlZahrani, Halal Alutaibi, Bayan Al Shuhayb, Tamim Alsultan, Hanan A Albenayyan, Bashayer F Al Furaikh

**Affiliations:** 1 Surgery, King Faisal University, Hofuf, SAU; 2 College of Medicine, King Faisal University, Hofuf, SAU; 3 Medicine, King Faisal University, Hofuf, SAU

**Keywords:** dry eye, digital screens, digital eye strain, covid- 19, ophthalmology

## Abstract

Background

Digital eye strain (DES) or computer vision syndrome (CVS) manifests as eye fatigue caused by prolonged exposure to screens and exaggerated by some attitudes. Online education plays a crucial role in helping schools, instructors, and universities ensure the continuity of the education process during the COVID-19 pandemic. There is a lack of attention given to the effects of online teaching on teachers’ eyes health during the pandemic and is nearly nonexistent. Hence, we aim to evaluate this among teachers in the Eastern Province of Saudi Arabia.

Methodology

A cross-sectional electronic self-administered questionnaire was distributed through social media applications among teachers in the eastern province of Saudi Arabia. The survey contained three main parts: biographical data, educational status, and eye health scale before and during the pandemic. All statistical analysis was done using IBM SPSS version 22 (IBM Corp., Armonk, NY).

Results

A total sample of 301 teachers was identified with ages ranging from 22 to 60 years, the majority were female (75.4%). Twenty-four point nine percent (24.9%) of the sample have a chronic disease, and 17.3% had previous LASIK surgery. Twenty-four point nine percent (24.9%) spent two to five hours teaching before the pandemic versus 60.8% with online education during the pandemic spent two to five hours daily. Fifty-two point eight percent (52.8%) of the teachers kept the distance between them and the digital screen at less than 50 cm. Eighty-one point four percent (81.4%) of teachers reported severe to moderate effects of online teaching using a computer/tablet/phone on their eye health. Fifty-two point two percent (52.2%) reported headache.

Conclusion

There is an obvious negative effect reflected by subjects’ symptomatology and complaints in their eyes. This should prompt health authorities to provide better teaching equipment and accessibility to essential eye care to teachers.

## Introduction

Since December 2019, the world has experienced a rapid spread of the new coronavirus disease 2019 (COVID-19) caused by severe acute respiratory syndrome coronavirus (SARS-CoV-2), a variant of coronavirus [1]. The World Health Organization (WHO) declared a pandemic on March 11, 2020. Hand contact or inhalation of viral droplets are the main transmission routes of coronavirus, which resulted in the rapid spread of the disease. Therefore, hygiene and social distancing are the main preventive methods to control the pandemic [2]. Many governments worldwide have taken severe measures to prevent the transmission of the virus. In particular, since February 2020, 191 countries have initiated the initial steps to suspend the educational institutions, including schools and universities, as part of these measures, and classroom teaching was replaced by online education-training activities [3].

Online education plays a crucial role in helping schools, instructors, and universities ensure the continuity of the education process during the COVID-19 pandemic. However, even though online education reduces transmission rates, it may harm eye health. Both students and the teaching faculty were left in front of screens for long periods during this process. These devices inflict damage by releasing short, high-energy waves that can penetrate the eyes and ultimately damage the retinal cells photochemically. Therefore, it makes a person more susceptible to eye problems, ranging from dry eyes to age-related macular degeneration. Collectively, it is referred to as digital eye strain (DES) or computer vision syndrome [4-5].

The American Optometric Association defines digital eye strain (DES) or computer vision syndrome as a wide range of visual and ocular symptoms caused by eye fatigue from prolonged exposure to screens [6]. Eye fatigue or computer vision syndrome (CVS) encompasses various symptoms such as eye discomfort, blurred vision, headache, lacrimation, dryness, redness, inability to focus, and neck and shoulder pain [7]. If a person spends more than two hours per day in front of a computer screen, he is more prone to develop those CVS symptoms [8-9]. CVS is a severe condition that affects attention and daily performance. In addition, CVS is caused by a reduced blinking reflex when focusing on a screen, resulting in exaggerated dry eyes, which is considered a significant contributor to the symptoms [10-12]. Other factors, such as usage duration, number of breaks, screen brightness, distance from the screen, and sitting posture, have been addressed in previous studies and established as risk factors for CVS [10,13].

Before COVID-19, many studies have found that CVS is common among office workers in general. For example, in 2016, one study discovered a prevalence of 67.4% among computer office employees, with females being more susceptible to the disease [14]. In addition, at the time of the pandemic, several publications showed increased rates of CVS-associated symptoms among the general population, children, and students in particular with similar associated risk factors such as increased computer usage rate per day, decreased screen distance, and pre-existing eye disease [15-19].

With the conversion of all the teaching classrooms to online classes, it is expected to have increased rates of eye fatigue or CVS among both students and teachers. However, to the best of our knowledge, the literature concerning the effect of online teaching on teachers’ eye health during the pandemic is not having enough attention and is nearly nonexistent. Therefore, the study aims to measure the effect of online education on the eye health of teachers and identify the possible risk factors of eye fatigue among them. The findings of this study will aid in recognizing the issue, raising awareness about it in their daily practices, and implementing prevention steps to reduce CVS-related symptoms.

## Materials and methods

Study design and participants

 A cross-sectional study was carried out in the eastern province of Saudi Arabia, from October 17, 2021, to February 20, 2022, to evaluate the effect of online education on eye health among Saudi teachers in the eastern province of Saudi Arabia. The minimum sample size was determined by the Richard Geiger equation to be 385 with a 95% confidence interval and a 5% margin of error. Both genders of Saudi teachers who participated in online teaching during the pandemic in the Eastern Province were included. Any participant who was not a teacher from the Eastern Province or did not complete all questions was excluded.

Data collection method

 An electronic self-administered questionnaire was distributed through social media applications such as WhatsApp. The questionnaire used in this study is self-structured with the revision of multiple ophthalmologists and general physicians to cover research objectives. The questionnaire is composed of three parts:

1. The first part contained demographical data, including age and gender.

2. The second part contained the educational status concerned with collecting data related to the academic status, specialty, teaching methods during the pandemic, the platform used during the pandemic, and the online daily hours spent before and during the pandemic.

3. The third part was contained the eye health scale in the pandemic concerned with collecting data related to eye health regarding the use of online teaching, associated symptoms and their frequency and intensity, and situational assessment, including blinking changes, distance from the screen, screen brightness, and eye gaze position.

Statistical Analysis 

After data extraction, they were revised, coded, and fed to statistical software IBM SPSS version 22 (IBM Corp., Armonk, NY). All statistical analysis was done using two-tailed tests, and a P-value less than 0.05 was statistically significant. Descriptive analysis was done based on the frequency and percent distribution for all variables, including teachers' personal data, ophthalmic history, online teaching, and related positions with the effect of online teaching on teachers' eye health and related symptoms. Cross-tabulation was used to assess the distribution of the eye health effects of online teaching during the COVID-19 pandemic among study teachers, their personal data, medical data, and online teaching data. The significance of relations was assessed using the Pearson chi-square test and exact probability test for small frequency distributions.

Ethical Considerations

After fulfilling all the needed ethical issues, ethical approval was obtained by the Institutional Research Board (IRB) and the Research Ethics Committee of King Faisal University in Al-Ahsa. The questionnaire contained a brief explanation of the aims of the study. Informed consent of all participants was taken before data collection.

## Results

A total sample of 301 teachers fulfilling the inclusion criteria completed the study questionnaire. Teachers’ ages ranged from 22 to 60 years, with a mean age of 38.6 ± 12.7 years. About 227 (75.4%) teachers were females. As for chronic health problems, 26 (8.6%) had diabetes mellitus (DM), 18 (6%) were hypertensive, and 31 (10.3%) had other sporadic health problems. A total of 221 (73.4%) had no chronic health problems. Also, 52 (17.3%) of the schoolteachers had previously undergone LASIK surgery while 240 (79.7%) had no history of eye surgery (Table 1).

**Table 1:** Bio-demographic data of sampled teachers for online learning, Eastern region, Saudi Arabia DM: diabetes mellitus; HTN: hypertension

Bio-demographic data	No	%
Age in years		
22-35	38	12.6%
36-50	229	76.1%
51-60	34	11.3%
Gender		
Male	74	24.6%
Female	227	75.4%
Had chronic health problems?		
DM	26	8.6%
HTN	18	6.0%
Asthma	5	1.7%
Others	31	10.3%
None	221	73.4%
Have you ever had eye surgery?		
LASIK	52	17.3%
Others	9	3.0%
No	240	79.7%

Table 2 shows the ophthalmic history among teachers in the Eastern region, Saudi Arabia. One-hundred seventy-three (57.5%) of the school teachers had previously visited an ophthalmologist. The leading causes for this visit were the correction of refractive errors (74%), followed by eye dryness (6.9%), eye allergy with itching (6.4%), blurred vision (7.5%), and 9.2% for other reasons.

**Table 2:** Ophthalmic history among teachers in the Eastern region, Saudi Arabia

Ophthalmic history	No	%
Have you ever visited an ophthalmologist?		
Yes	173	57.5%
No	128	42.5%
Causes of visiting ophthalmologist		
Refractive error	128	74.0%
Dryness	12	6.9%
Lacrimation with itching	11	6.4%
Blurred vision	13	7.5%
Others	16	9.2%
Have you ever used moistening eye drops?		
Yes	48	52.2%
No	44	47.8%
Have you ever had eye surgery?		
LASIK	52	17.3%
Others	9	3.0%
No	240	79.7%

Table 3 shows the online teaching provided by school teachers during the COVID-19 pandemic in the Eastern region, Saudi Arabia. As for teaching methods during the COVID pandemic, online learning with interactive participation was the most used method (82.1%), followed by online learning with uploading materials (54.2%), online learning without interactive participation (16.9%), and using virtual classrooms with Microsoft PowerPoint (2.3%; Microsoft Corporation, Redmond, WA). Regarding the used platforms, Microsoft Teams (Microsoft Corporation) was used by 83.4% of the teachers, followed by Zoom (25.2%; Zoom Video Communications, San Jose, California), the platform (12%), and Classera (5.3%; San Francisco, CA). About daily hours spent on online teaching before the COVID pandemic, 13% spent about one hour daily, 24.9% spent two to five hours, 15.6% spent more than five hours, while 46.5% never used online learning before the pandemic. About daily hours spent on online teaching after the covid pandemic, 60.8% spent two to five hours, 35.2% spent more than five hours, and only 4% spent one hour daily. 

**Table 3:** Online teaching provided by the study teachers during the COVID-19 pandemic, Eastern region, Saudi Arabia

Online teaching	No	%
Teaching methods during theCOVID pandemic		
Online learning with interactive participation	247	82.1%
Online learning with uploading materials	163	54.2%
Online learning without interactive participation	51	16.9%
Using virtual classrooms with PowerPoint	7	2.3%
Displaying videos, games, and competitions	1	.3%
What was the used platform		
Microsoft Teams	251	83.4%
Zoom	76	25.2%
The platform	36	12.0%
Classera	16	5.3%
Webex	6	2.0%
Daily hours spent on online teaching before the COVID pandemic		
1 hour	39	13.0%
2-5 hours	75	24.9%
> 5 hours	47	15.6%
Not used	140	46.5%
Daily hours spent on online teaching after the COVID pandemic		
1 hour	12	4.0%
2-5 hours	183	60.8%
> 5 hours	106	35.2%

Table 4 shows the teachers' positions and distance during online learning during the COVID-19 pandemic, in the Eastern region, Saudi Arabia. About 52.8% of the teachers keep distance between them and the digital screen display while using the device of less than 50 cm while 43.2% keep a distance of 50 cm. As for screen brightness while using the device, 75.4% use average brightness, only 6.3% use low brightness, and 18.3% use high/very high brightness. Considering the position of the eyes towards the screen, it was straight ahead among 68.8% users, down a little among 28.9%, and a little higher among 2.3%.

**Table 4:** Teachers' positions during online learning with the COVID-19 pandemic, Eastern region, Saudi Arabia

Online learning position	No	%
The distance between you and the digital screen display while using the device		
< 50 cm	159	52.8%
50 cm	130	43.2%
> 50 cm	12	4.0%
Screen brightness while using the device		
Low	19	6.3%
Normal	227	75.4%
High	42	14.0%
Very high	13	4.3%
The position of the eyes toward the screen		
Down a little	87	28.9%
Straight ahead	207	68.8%
A little higher	7	2.3%

Table 5 shows the teachers' eye health with online learning during the COVID-19 pandemic, in the Eastern region, Saudi Arabia. About 84 (27.9%) teachers reported the severe effect of online teaching using a computer/tablet/phone on their eye health, while 161 (53.5%) had a moderate effect and 56 (18.6%) had no effect. As for eye symptoms related to dryness, 43.5% complained of a feeling of stinging, burning, or roughness in the eyes, followed by eye redness (39.2%), excessive lacrimation (34.6%), blurred vision (34.6%), photosensitivity (32.9%), feeling of having a foreign body in the eyes (29.2%), difficulty driving at night (10.6%), sticky discharge in or around the eyes, and difficulty wearing contact lenses (4% each). Regarding myopia symptoms, the headache was the most reported (52.2%), followed by difficulty concentrating (34.9%), pain and fatigue in the eyes, especially when looking at distant objects and staring (31.9%), blurred vision (26.9%), and diplopia (10%). As for symptoms frequency, 127 (55.7%) had these symptoms rarely or once a week. Symptoms were mild among 31.6% of the teachers, moderate among 45.2%, and severe among 23.2%. Almost 29.9% of the teachers noticed increased changes in blinking during the pandemic while 57.5% had no change but decreased among 12.6%.

**Table 5:** Teachers' eye health with online learning during the COVID-19 pandemic, Eastern region, Saudi Arabia

Eye health	No	%
Effect of online teaching using computer/tablet/phone on your eye health		
Severe effect	84	27.9%
Moderate effect	161	53.5%
No effect	56	18.6%
Eye symptoms related to dryness		
None	48	15.9%
A feeling of stinging, burning, or roughness in the eyes	131	43.5%
Eye redness	118	39.2%
Excessive lacrimation	104	34.6%
Blurred vision	104	34.6%
Photosensitivity	99	32.9%
Feeling of having feedback in the eyes	88	29.2%
Difficulty driving at night	32	10.6%
Sticky pus in or around the eyes	12	4.0%
Difficulty wearing contact lenses	12	4.0%
Myopia symptoms		
None	73	24.3%
Headache	157	52.2%
Difficulty concentrating	105	34.9%
Pain and fatigue in the eyes, especially when looking at distant objects and staring.	96	31.9%
Blurred vision	81	26.9%
Diplopia	30	10.0%
Frequency of these symptoms		
Sometimes (rarely or once/week)	127	55.7%
Usually (daily to 3 times/week)	101	44.3%
Severity of these symptoms		
Mild	72	31.6%
Moderate	103	45.2%
Severe	53	23.2%
Have you noticed changes in your eyelashes "blink" during the pandemic		
Increased	90	29.9%
Decreased	38	12.6%
No change	173	57.5%

Table 6 shows the eye health effect of online learning on teachers’ demographic, teaching, and position. A moderate to severe effect on eye health was reported by 82.4% of teachers aged 51-60 years compared to 63.2% of those aged 22-35 years with recorded statistical significance (P=.001). Also, 87.7% of female teachers had moderate to severe effects compared to 75% of males (P=.001). Spending more than five hours on online learning before the COVID-19 pandemic was associated with moderate to severe effects on 87.2% of teachers’ eye health versus 89.7% of those who spent one hour (P=.005). During the pandemic, spending more than five hours was associated with moderate to severe effects on the eye health of 89.6% of teachers compared to 75% of those who spent 1 hour (P=.003). Almost 85.5% of teachers who previously visited ophthalmologists had moderate to severe effects on their eye health due to online learning during the COVID-19 pandemic versus 75.8% of others who did not (P=.004).

**Table 6:** Effect of online learning on eye health based on teachers’ demographic data, teaching data, and position

Factors	Effect of online teaching using computer/tablet/phone on your eye health						p-value
	Severe effect		Moderate effect		No effect		
	No	%	No	%	No	%	
Age in years							.001*
22-35	1	2.6%	23	60.5%	14	36.8%	
36-50	74	32.3%	119	52.0%	36	15.7%	
51-60	9	26.5%	19	55.9%	6	17.6%	
Gender							.001*
Male	11	14.9%	35	47.3%	28	37.8%	
Female	73	32.2%	126	55.5%	28	12.3%	
Had chronic health problems?							.175
Yes	23	28.8%	37	46.3%	20	25.0%	
No	61	27.6%	124	56.1%	36	16.3%	
Daily hours spent on online teaching before the COVID pandemic							.005*
1 hour	12	30.8%	23	59.0%	4	10.3%	
2-5 hours	11	14.7%	40	53.3%	24	32.0%	
> 5 hours	19	40.4%	22	46.8%	6	12.8%	
Not used	42	30.0%	76	54.3%	22	15.7%	
Daily hours spent on online teaching after the COVID pandemic							.003*^$^
1 hour	3	25.0%	6	50.0%	3	25.0%	
2-5 hours	38	20.8%	103	56.3%	42	23.0%	
> 5 hours	43	40.6%	52	49.1%	11	10.4%	
The distance between you and the digital screen display while using the device							.082^$^
< 50 cm	54	34.0%	80	50.3%	25	15.7%	
50 cm	29	22.3%	74	56.9%	27	20.8%	
> 50 cm	1	8.3%	7	58.3%	4	33.3%	
Screen brightness while using the device							.170^$^
Low	3	15.8%	11	57.9%	5	26.3%	
Normal	63	27.8%	116	51.1%	48	21.1%	
High	14	33.3%	26	61.9%	2	4.8%	
Very high	4	30.8%	8	61.5%	1	7.7%	
The position of the eyes toward the screen							.648
Down a little	28	32.2%	42	48.3%	17	19.5%	
Straight ahead	53	25.6%	116	56.0%	38	18.4%	
A little higher	3	42.9%	3	42.9%	1	14.3%	
Have you ever visited an ophthalmologist?							.004*
Yes	60	34.7%	88	50.9%	25	14.5%	
No	24	18.8%	73	57.0%	31	24.2%	
Have you ever used moistening eye drops?							.847
Yes	10	20.8%	31	64.6%	7	14.6%	
No	10	22.7%	26	59.1%	8	18.2%	
Have you ever had eye surgery?							.273^$^
LASIK	14	26.9%	29	55.8%	9	17.3%	
Others	1	11.1%	8	88.9%	0	0.0%	
No	69	28.8%	124	51.7%	47	19.6%	

## Discussion

The COVID-19 pandemic has forced schools and universities worldwide to switch to online learning. Moreover, this was implemented to reduce the disease spreading among students, teachers, and the entire community. Online teaching has shown to be of great help to schools and universities during the COVID-19 pandemic. Prolonged hours spent on the device screen can harm the visual feedback process and posture control system, which will affect eye health.

Moreover, during the home quarantine, the entertainment method for most of the society was by using screens or devices, whether to watch the news, watch series and movies, or even play digital games for long hours, which led to an increased risk of eye strain and fatigue. Eye fatigue has a wide range of visual symptoms like burning eyes, blurred vision, difficulty concentrating, tiredness, headaches, and soreness of the eyes [20]. 

In this study, the investigation of the effect of online education on eye health was detected by using an online education eye health scale and eye fatigue scale, which showed that online education harmed the eye health of teachers.

In the current study, the participants were 301, and the result showed the majority effect on the female gender with 87.7% compared with the male gender with 75%. Also, there is a moderate to severe effect on eye health reported by 82.4% of teachers aged 51-60 years compared to 63.2% of those aged 22-35 years.

Before the COVID-19 pandemic, this research found that most schoolteachers with a percentage of 46.5% had never used the online way of teaching. Moreover, during the pandemic, most schoolteachers have spent two to five hours daily on online teaching, with a percentage of 60.8%. Tawil et al. reported the use for more than five hours as a significant risk factor for digital eye strain [21]. On the other hand, Ahuja et al. stated that a digital device used for more than five years with six hours of continuous use is a significant risk factor for eye strain, and subjects in this study have spent less than three years of continuous use of digital screens with the majority spending two to five hours only [22].

Other factors contributing to the effect of screens on the eyes are, first, the distance between the eyes and the screen, where it was found that most schoolteachers, with a percentage of 54.8%, kept a close distance (less than 50 cm). The recommended screen distance by the American Optometric Association is 51 cm. Second, the American Optometric Association recommended the screen's brightness to be adjusted to around 100-150 cd/m2, and this research has found that most schoolteachers use average brightness. Third, the position of the eyes on the screen can have a considerable effect, and in this study, it was found that 68.8% have a straight-ahead position. Although the position recommended by the American Optometric Association is to adjust the monitor height so that the top of the screen is at or slightly below the eye level, Ahuja et al. have found that when the device is placed below the eye level, it causes more symptoms that accommodations and convergence might cause during near vision. While in this research, the majority had a straight-ahead position; this is in contrast to the observation made by Agarwal et al., where the eyes were placed above the screen level to decrease the complaints of eye strain [22].

As for the prior mentioned factors, the effect on the eyes can be significant. This study found that most schoolteachers (53.5%) had a moderate effect on their eye health. Most subjects have complained of stinging, burning, roughness of eyes and eye redness, excessive lacrimation, and blurring of vision with photosensitivity, which suggests eye dryness. Similar symptoms were found in a recent study by Nawaf Al Marzouki et al., who assessed the digital eye strain during COVID-19 lockdown in Jeddah [15]; this is in contrast to Tawil et al., who reported the shoulder and neck pain to be the most common complaint [21]. Ahuja et al. found the burning sensation of the eyes, dryness of eyes, watering, and itching to be the most common complaints [22].

Moreover, 52.2% of schoolteachers have reported headaches, which may suggest myopia. Regarding the frequency of symptoms, the majority rarely had the symptoms, with a percentage of 55.7%. However, 45.2% when having the symptoms grade it as moderate.

In a 2016 study by Bostanci [23], exposure to digital screens worsened eye symptoms, and in a study on computer use and vision, Shantakuri reported that new eye symptoms appeared as time spent on digital screens increased, the most common are redness and burning [24]. In this study, 301 teachers developed new symptoms after introducing online education. The most common occurrences were 34.6% excessive tearing of the eyes, 34.6% blurred vision, and 32.9% photosensitivity.

The results showed higher differences in gender for headaches, which indicates that female teachers are at increased risk. Furthermore, this is consistent with studies showing a higher score of somatic complaints in females [25]. On the other hand, the incidence of headache has been noted to have a significant relation with distance, especially if it is less than 50 cm; this is consistent with some studies highlighting that visual fatigue increases with shorter distances [26-27].

Regarding blinking, the results showed that 29.9% of teachers had an increased change in blinking during the pandemic while 57.5% had no change. Furthermore, blinking decreased among 12.6%, suggesting the continuous use of smartphones causes dry eye-related symptoms. When relating dry eye and age between our study and the literature, 43.5% complained of a feeling of dryness of the eye, and it has a significant association with increased age and corresponds to the prior study's findings [28-30]. On the other hand, the dryness of the eye shows a higher score among females, which correlates with other studies [30].

## Conclusions

Our study shows a consensus on the importance and value of eye health and the effects of online teaching during the COVID-19 pandemic. There is a noticeable effect reflected in subjects’ symptomatology and complaints. This should prompt health authorities to provide better teaching equipment and accessibility to essential eye care gadgets to teachers. Furthermore, our study shows that increased online teaching hours, posture, and screen distance are the most influential factors in teachers’ eye health. Future studies should aim to understand and compare the effects of online and on-site teaching in different areas and on the national level.

## Appendices

The Effect of Online Education on Healthy Eyes of Saudi Teachers in the COVID-19 Pandemic: A Local Study

We are a group of medical students at King Faisal University under the supervision of the Department of Ophthalmology. We aim to study the effect of distance education on eye health for female teachers and educators in the Eastern Province during the Corona pandemic.

This research targets all teachers and teachers who have taught remotely.

The information provided will be strictly confidential and your participation in this survey is optional

Personal information

1. Gender

·      Male

·      feminine

2. Age

·      22 - 35

·      35 - 50

·      50 - 60

·      older than 61

3. Place of residence

·      Dammam

·      Alkhobar

·      Jubail

·      Dhahran

·      Qatif

·      Khafji

·      Alnaria 

·      Ras Tanoura 

·      Alashsa

·      Hafar Al-Batin

·      Other: …………………..

Educational Status

4. What is your current employment status?

·      Class teacher

·      Retired teacher

·      Head master 

·      Administrative

5. What is your speciality? Please select all that apply

·      Mathematics

·      Science 

·      Chemistry

·      Physics

·      Islamic

·      Arabic

·      Physical education

·      Family education

·      Art

·      Other: ………………………

6. What teaching methods have you used throughout the pandemic? Please select all that apply

·      Online lessons, without interactive participation

·      Online lessons with interactive participation

·      Online lessons with downloaded educational materials

·      Other: ………………………………………….

7. If you were using a web-based platform for interactive presentations during a pandemic, which one did you use? Please select all that apply

·      Zoom 

·      WebEx

·      Microsoft Teams

·      Almanasa 

·      Classera 

·      Others: ………………………… 

8. How many daily hours did you spend teaching students online “before” the pandemic?

·      I did not use online education

·      Hour

·      Two to five hours

·      More than five hours

9. How many daily hours do you spend teaching students online “during” the pandemic?

·      Do not use online education

·      Hour

·      Two to five hours

·      More than five hours

Eye health scale with distance education during the corona pandemic

10. How did your distance education on your computer/tablet/mobile phone affect your eye health during the pandemic?

·      My eye health has not changed

·      Slight deterioration in my eye health

·      Severe deterioration in my eye health

Accompanying symptoms:

11. Please clarify whether you suffer from any of the symptoms of dry eyes. Please select all that apply

·      There are no symptoms

·      A feeling of stinging, burning, or roughness in the eyes

·      Sticky discharge in or around the eyes

·      Photosensitivity

·      Eye redness

·      Feeling of having something in the eyes

·      Difficulty wearing contact lenses

·      Difficulty driving at night

·      Watery eyes

·      Blurred vision

·      Others: ……………………………………..

12. Please indicate if you suffer from any of the symptoms of near-sightedness. Please select all that apply

·      There are no symptoms

·      Blurred vision

·      Headache

·      Double vision

·      Pain and fatigue in the eyes, especially when looking at distant objects and staring

·      Difficulty concentrating

·      Other: ……………………………

13. If you have symptoms, determine the frequency of symptoms, considering the following:

·      Never = No symptoms at all

·      Sometimes = Sporadic episodes or once a week

·      Often or always = 2 or 3 times a week or almost every day

14. If you have symptoms, determine the severity of the symptoms

·      Light

·      Moderate

·      Intense

15. Have you noticed changes in your blinking during the pandemic?

·      Increased

·      Did not change

·      Decreased 

16. What is the distance between you and the digital screen while using the device (50 cm is approximately the length of the arm.)

·      cm 50

·      Less than 50 cm - very close to you

·      More than 50 cm - too far from you

17. How would you rate the screen brightness while using the device

·      Very bright

·      Glare

·      Natural

·      Matt

·      Very matte

18. How do you evaluate the position of your eyes towards the screen?

·      Down a little

·      Straight ahead

·      Up a little

19. Have you ever visited an ophthalmologist?

·      Yes

·      No 

20. If the answer to the previous question is yes. Please specify the reason for visiting the doctor

·      Refractive errors (long-sightedness / short-sightedness)

·      Age-related macular degeneration

·      Cataract 

·      Diabetic retinopathy

·      Glaucoma

·      Other:……………………

21. Have you ever used eye drops

·      Yes

·      No 

22. Do you have chronic diseases?

·      No 

·      Obesity

·      Diabetes

·      Hypertension 

·      Cholesterol

·      Other: …………………….

23. Have you ever used medication for a long period of time?

·      No 

·      Isotretinoin capsules (Roaccutane)

·      Diabetes treatments

·      Hypertension treatments

·      Others:……………..

24.  Have you ever had eye surgery?

·      No

·      LASIK

·      Laser

·      Cataract surgery

-      Glaucoma surgery
